# Dorsolumbar Junction Spinal Tumors at a Tertiary Care Center in Pakistan: Neurofibroma Predominance and Surgical Outcomes

**DOI:** 10.12669/pjms.41.13(PINS-NNOS).13430

**Published:** 2025-12

**Authors:** Talha Sajid, Adeel-Ur-Rehman, Samra Majeed, Sikander Ali Dheraj, Ansarullah Khan, Nasruddin Ansari

**Affiliations:** 1 Talha Sajid, MBBS. Punjab Institute of Neurosciences, Lahore, Pakistan; 2 Adeel-Ur- Rehman, MBBS. Punjab Institute of Neurosciences, Lahore, Pakistan; 3 Samra Majeed, MBBS, FCPS. Punjab Institute of Neurosciences, Lahore, Pakistan; 4 Sikandar Ali Dheraj, MBBS, MS. Punjab Institute of Neurosciences, Lahore, Pakistan; 5 Ansarullah Khan, MBBS. Punjab Institute of Neurosciences, Lahore, Pakistan; 6 Nasruddin Ansari, MBBS. Punjab Institute of Neurosciences, Lahore, Pakistan

**Keywords:** Spinal Neoplasms, Spinal Cord Neoplasms, Intradural Extramedullary, Neurofibroma, Laminectomy, Pakistan

## Abstract

**Objective:**

To estimate the prevalence and evaluate the surgical outcomes of dorsolumbar junction spinal tumors at a tertiary care center in Pakistan.

**Methodology:**

This retrospective cohort study was conducted at Punjab Institute of Neurosciences, Lahore, including 35 patients operated for dorsolumbar junction (D11–L2) tumors between January 2021 and October 2024. Data extracted from records and PACS included demographics, presentation, imaging, operative details, histopathology, extent of resection, and outcomes. Tumors were classified by compartment and resection status, and primary outcomes were symptomatic improvement and complications. Data were analyzed descriptively using Microsoft Excel.

**Results:**

The cohort comprised patients with a mean age of 36.25 ± 16.7 years, with male predominance (54.3%, n=19). Intradural extramedullary tumors were most prevalent (80.0%, n=28), with neurofibromas being the most common histological type (51.4%, n=18). Predominant presenting symptoms included backache radiating to lower limbs (80.0%, n=28) and neurological deficits. A posterior surgical approach was used in all patients achieving gross total resection(GTR) and near total resection (NTR) in 85.7% of patients. Immediate symptom relief was reported in 57.1% (n=20) of patients, with a tumor recurrence rate of 8.6% (n=3). Complications included new neurological deficits (5.7%, n=2) and cerebrospinal fluid leakage (5.7%, n=2).

**Conclusion:**

This study highlights that dorsolumbar junction tumors in our Pakistani cohort were predominantly intradural-extramedullary, mainly neurofibromas and schwannomas, most often at D12–L1/L1–L2. Standard posterior approaches achieved effective resections with low complications and early symptomatic improvement. These findings demonstrate that with modern imaging and surgical expertise, centers in Pakistan can deliver care comparable to international standards, while larger multicenter studies are needed to validate and extend these results.

## INTRODUCTION

The dorsolumbar junction is a critical anatomical region where various spinal tumors can manifest, presenting unique clinical challenges and requiring tailored surgical approaches. Spinal tumors in this area can arise from different tissues, including the vertebrae, spinal cord, and surrounding soft tissues, and can be either primary or metastatic in origin. Accurate differentiation between these entities is crucial, as treatment strategies vary significantly; while some conditions may require surgical intervention, others can be managed conservatively.^1^

Radiological imaging plays a pivotal role in the initial assessment and ongoing management of spinal tumors. Magnetic resonance imaging (MRI) is the gold standard for visualizing spinal lesions, providing detailed information about the tumor’s location, size, and relationship to surrounding structures.^2^ MRI findings can help distinguish between benign and malignant tumors, as well as identify the presence of associated edema or other complications.^3^ However, the interpretation of these imaging studies must be correlated with clinical history and symptoms to ensure accurate diagnosis.^4^ For instance, certain benign conditions, such as lumbar disc sequestration can mimic the appearance of tumors on imaging, leading to potential misdiagnosis and inappropriate treatment.^3^

Surgical management of spinal tumors at the dorsolumbar junction requires a multidisciplinary approach, often involving neurosurgeons, orthopedic surgeons, and radiologists. The choice of surgical technique depends on various factors, including the tumor’s histological type, location, and the patient’s overall health status.^5^,^6^ Recent advancements in minimally invasive surgical techniques, such as unilateral bi-portal endoscopic tumor removal, have shown promise in improving patient outcomes while minimizing postoperative complications.^7^ These techniques allow for effective tumor resection with reduced recovery times, making them particularly beneficial in a tertiary care setting where patient turnover is high.

Histopathological evaluation remains essential for confirming the diagnosis of spinal tumors and guiding treatment decisions. Tumors such as ependymomas, meningiomas, and schwannomas have distinct histological features that can influence prognosis and treatment options.^5^,^8^ For example, spinal ependymomas, which account for a significant proportion of spinal tumors, can vary in their clinical behavior based on their histological grade.^4^,^8^ Extradural spinal tumors are mostly metastatic (90%), primarily from the lung, breast, prostate, and kidney. Primary extradural tumors (10%) include benign lesions such as neurofibromas, meningiomas, angiolipomas, and aneurysmal bone cysts, and malignant tumors like Ewing’s sarcoma, chordoma, plasmacytoma, and neuroblastoma.^6^ Accurate histopathological diagnosis is crucial, as some tumors may respond well to non-surgical treatments such as chemotherapy or radiation therapy, while others necessitate surgical intervention.^9^,^10^

Despite global advancements in the diagnosis and management of spinal tumors, there is limited region-specific data from Pakistan, particularly concerning tumors at the dorsolumbar junction (D11–L2), a biomechanically and neurologically critical area. Existing literature often generalizes spinal tumors without focusing on this anatomically distinct zone, resulting in a gap in understanding regarding their unique clinical presentation, imaging characteristics, surgical challenges, and outcomes in local populations. This study attempted to address that gap by evaluating the clinico-radiological features, surgical approaches, and postoperative outcomes of dorsolumbar junction tumors, while laying the groundwork for future research in spinal oncology within similar healthcare environments.

## METHODOLOGY

This study was designed as a retrospective cohort conducted at the Department of Neurosurgery, Unit-III, Punjab Institute of Neurosciences (PINS), Lahore. All consecutive patients who underwent surgery for spinal tumors localized to the dorsolumbar junction (D11–L2) between January 2021 and October 2024 were included, and data were analyzed in January–February 2025. A non-probability convenience sampling method was applied, resulting in a total sample of 35 patients.

### Ethical Approval:

Exemption was obtained from the Institutional Review Board of Punjab Institute of Neurosciences, Lahore, under reference no. 2042/IRB/PINS/Approval/2025, dated 30-01-2025.

### Inclusion Criteria:

Consecutive patients of any age or sex who underwent surgical treatment at our center during the study period for spinal tumors centered at the dorsolumbar junction (D11–L2), including lesions that extended into this segment from adjacent levels as confirmed on MRI (and, where available, intraoperative findings) were included in the study.

### Exclusion Criteria:

Patients with lesions confined entirely outside the D11–L2 segment and patients managed non-operatively during the study period were not included in the study.

Clinical, radiological, operative, and postoperative information was collected from hospital records, including patient files and medical registration numbers. Imaging data were retrieved through the Picture Archiving and Communication System (PACS). A structured Google Form was used to enter patient details, and all entries were cross-checked with operative notes and PACS reports to minimize transcription errors. The variables collected included demographics, presenting complaints, neurological status, comorbidities, tumor level, radiological compartment, operative approach, surgical duration, blood loss, fixation requirement, histopathology, extent of resection, and immediate postoperative outcomes including complications.

### Operational definitions:

The tumor compartment was classified as intradural extramedullary (IDEM), intramedullary, or extradural based on MRI findings corroborated by intraoperative confirmation. The extent of resection was defined as gross total resection (removal >99%), near-total resection (≥90%), or subtotal resection (50–90%). Levels were assigned within the anatomical boundaries of D11 to L2, and lesions contiguous with or extending into this region were included accordingly. Primary outcomes were defined as the degree of symptomatic relief after surgery, categorized as immediate, delayed, or absent, and the occurrence of postoperative complications such as new neurological deficits, cerebrospinal fluid leak, wound complications, or re-exploration. Secondary outcomes included extent of resection achieved, requirement for fixation, and early adjuvant therapy when applicable.

The data were compiled and analyzed using Microsoft Excel. Descriptive statistical methods were employed to summarize the findings, with means, standard deviations, medians, and frequency distributions calculated as appropriate. Given the relatively small sample size and descriptive aim of the study, no formal hypothesis testing was performed.

## RESULTS

The cohort included 35 patients with confirmed dorsolumbar junction (D11–L2) tumors, with a mean age of 36.25 years (SD ± 16.7 years) and a male predominance (54.3%, n=19). The most frequent presenting symptom was backache radiating to the lower limbs (80.0%, n=28), followed by neurological deficits including lower limb weakness (31.4%, n=11), numbness (22.9%, n=8), isolated backache (14.3%, n=5), and muscle atrophy (8.6%, n=3). Systemic comorbidities included hypertension (14.3%, n=5) and diabetes mellitus (8.6%, n=3).

**Fig.1:**
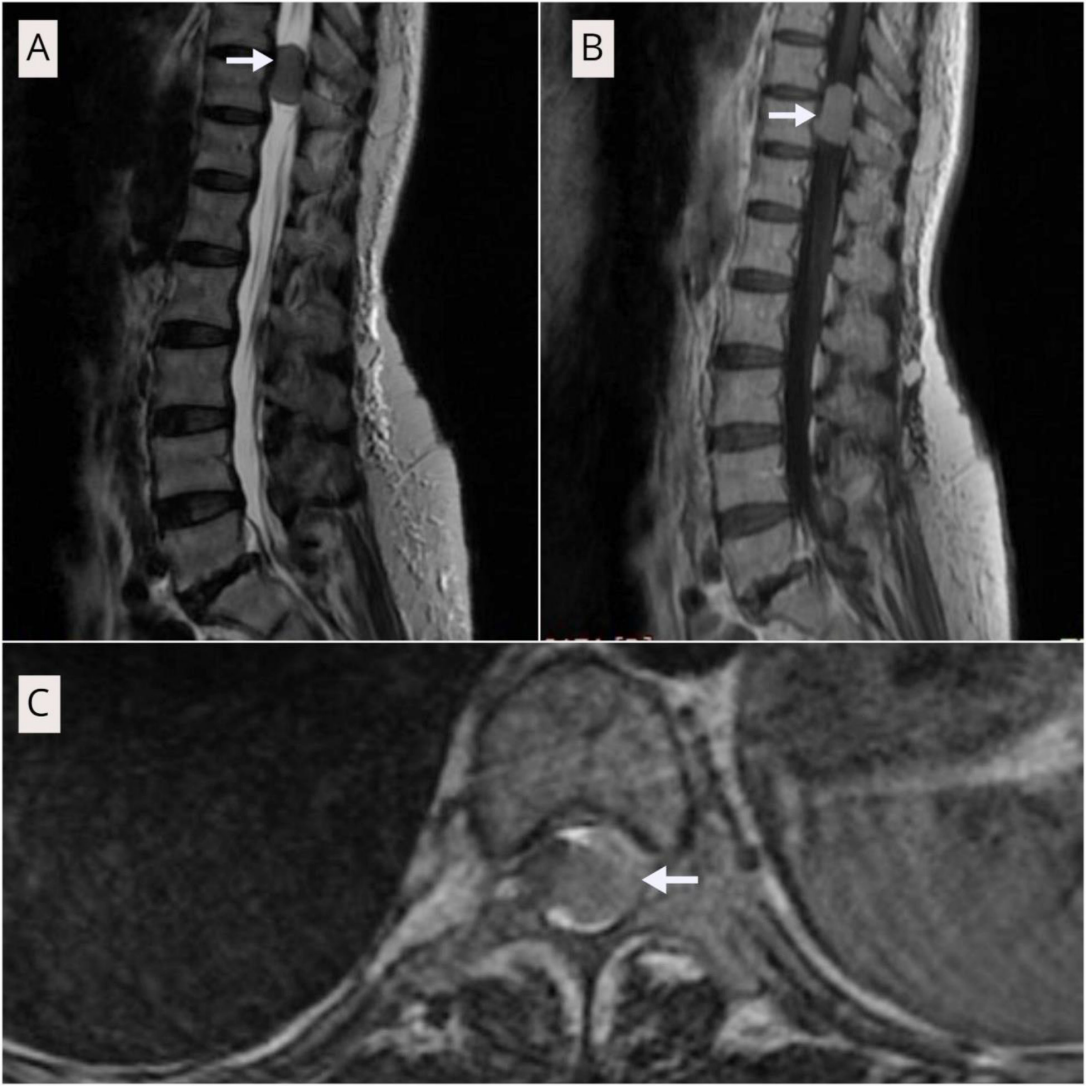
A: T2 weighted sagittal view of dorsolumber spine shows iso to hypointensity signal at D10-D11 vertebral level (white arrow). B: T1 post gadolinium contrast sagittal view of dorsolumber spine shows hyperintensity signal at D10-D11 vertebral level (white arrow). C: T1 post gadolinium contrast axial view of D11 vertebral level shows the tumor pushing the cord on the right side(white arrow).

MRI demonstrated intradural extramedullary (IDEM) tumors in 80.0% (n=28). On pre-contrast T1-weighted images, IDEM lesions were usually iso to mildly hypointense to the spinal cord; T2-weighted images were typically iso to hypointense. Post-contrast T1 with gadolinium showed avid enhancement generally homogeneous (Figure 1) and variable in nerve-sheath tumors. Intramedullary tumors (5.7%, n=2) were iso- to hypointense on T1, hyperintense on T2, with variable (often patchy) post-contrast enhancement. Extradural lesions (14.3%, n=5) displayed mixed T1/T2 signals with robust post-contrast enhancement. Tumors were most frequent at D12–L1 (57.1%, n=20), L1–L2 (28.6%, n=10) and the remainder (14.3%, n=5) extended into the dorsolumbar junction from levels above D11 or below L2.

**Fig.2:**
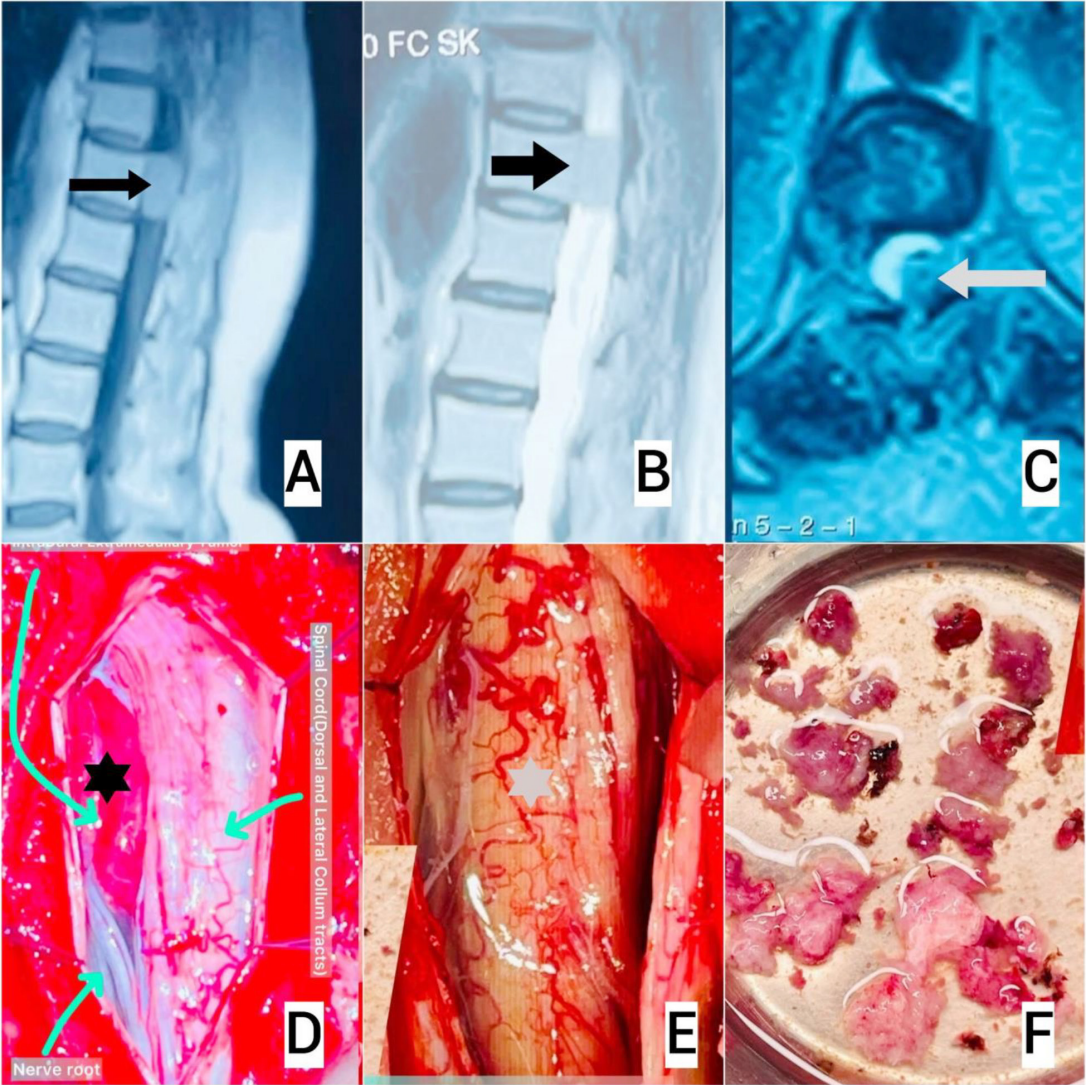
A:T1 post gadolinium contrast sagittal view of dorsolumber spine shows hyperintensity signal at D12 vertebral level (black arrow). B: T2 weighted sagittal view of dorsolumber spine shows isointensity signal at D12 vertebral level (black arrow). C: T2 weighted axial view of D12 vertebral level shows the tumor pushing the cord/conus on the right side(black arrow). D: Per-operative view of the extramedullary lesion attached with the dura compressing the conus/cord and nerve roots(black asterisk) E: After gross total resection, cord is free of any compression(grey asterisk). F: Multiple chunks of the tumor, tumor was soft in consistency and highly vascular.

All patients underwent posterior surgical approaches, with laminectomy and tumor excision performed in 94.3% (n=33) and biopsy alone in 5.7% (n=2) for highly vascular tumors or lesions adherent to critical nerve roots. The mean surgical duration was 145.6 ± 45.2 minutes, with an average intraoperative blood loss of 428.6 ± 127.3 mL. Resection outcomes included GTR and NTR was achieved in 85.7% (n=30) as shown in (Figure 2), subtotal resection (STR) in 8.6% (n=3) involving both intramedullary ependymomas, and biopsy-only in 5.7% (n=2). Spinal fixation was required in 8.6% (n=3).

Surgical Outcomes, postoperative recovery and complications are explained in Table-I. Histopathological results of these dorsolumbar junction tumors are listed in Table-II.

**Table-I:** Surgical Outcomes, Postoperative Recovery and Complications

Parameter	Category	Frequency (n=35)	Percentage (%)
Surgical Approach	Laminectomy & Tumor Excision	30	85.7%
	Biopsy Only	5	14.3%
Mean Surgical Duration	—	145.6 ± 45.2 min	—
Mean Blood Loss	—	428.6 ± 127.3 mL	—
Extent of Resection	Gross/Near Total Resection (GTR/NTR ≥90%)	30	85.7%
	Subtotal Resection (STR 50–90%)	3	8.6%
	Biopsy Only	2	5.7%
Spinal Fixation Required	Yes	3	8.6%
Symptom Relief	Immediate Improvement	20	57.1%
	Delayed Improvement	6	17.1%
	No Improvement	9	25.7%
Complications	New Lower Limb Weakness	2	5.7%
	CSF Leakage (Managed with re-exploration and repair of dural defect with fascial patch and sealant)	2	5.7%
	Wound Infection	1	2.9%
Tumor Recurrence	Associated with STR/Biopsy	3	8.6%

**Table-II:** Histopathological Distribution

Tumor Type	Frequency (n=35)
***Intradural Extramedullary (IDEM) Tumors***	
- Neurofibroma	51.4% (n=18)
- Schwannoma	20% (n=7)
- Meningioma	5.7% (n=2)
- Arachnoid Cyst	2.9% (n=1)
***Intramedullary Tumors***	
- Ependymoma	5.7% (n=2)
***Extradural Tumors***	
- Metastases	2.9% (n=1)
- Osteoblastoma	5.7% (n=2)
- Ewing’s Sarcoma	2.9% (n=1)
- Hemangioma	2.9% (n=1)

## DISCUSSION

The investigation of spinal tumors, particularly at the dorsolumbar junction, is critical due to their significant clinical implications and the diversity in histological presentations. The study cohort at our tertiary care facility revealed a predominance of intradural extramedullary tumors, comprising 80% of the cases. Within this subset, neurofibromas represented the most common histological type, accounting for over half of the cases, followed by schwannomas and meningiomas. These findings align with existing literature, showing that intradural extramedullary tumors, such as neurofibromas and schwannomas, are often prevalent in spinal regions susceptible to mechanical compromise and neurological deficits.^4^,^10^-^12^

The demographic profile of our cohort average age of 36.25 years with male predominance is noteworthy. This aligns with other studies that show male individuals are frequently affected by spinal tumors. Factors such as occupational hazards and activity levels may contribute to increased spinal stress and injury.^12^,^13^ Moreover, MRI has proven to be an essential diagnostic tool in identifying the exact location and type of tumors, reinforcing previous assertions about the efficacy of MRI scans in the context of spinal pathologies.^2^ Comprehensive imaging supports differentiation between various types of space-occupying lesions, allowing for timely and appropriate surgical interventions.^14^

Spinal neoplasms are categorized by MRI into three groups. Extradural tumors specifically metastatic lesions appear T1 hypointense, T2 hypointense on MRI, with contrast enhancement. Lytic lesions (lung, kidney, thyroid) show T2 hyperintensity, while blastic lesions (prostate) appear T1/T2 hypointense.^11^,^14^ IDEM tumors, mainly neurofibromas, schwannomas and meningiomas show distinct MRI patterns. Schwannomas are T1 isointense/hypointense, T2 hypointense with heterogeneous enhancement. Meningiomas appear T1 isointense, T2 iso-/hypointense, with homogeneous enhancement and a dural tail sign. Neurofibromas are fusiform, T2 hypointense, often showing a target sign. MRI remains key for localization and surgical planning.^11^ Intramedullary spinal tumors show distinct MRI features: astrocytomas appear infiltrative with T1 hypointensity, T2 hyperintensity, and heterogeneous enhancement, while ependymomas are well-circumscribed with T1 isointensity, T2 hyperintensity, and homogeneous enhancement, often with cysts. Hemangioblastomas present as hypervascular nodules with cystic components and flow voids, whereas epidermoid/dermoid cysts are hyperintense on T1/T2 with possible peripheral enhancement. MRI remains crucial for diagnosis, localization, and surgical planning.^2^,^11^

Surgical approaches to resection in our cohort predominantly featured the posterior approach, which consisted of laminectomy procedures. This method is often favored for its ability to provide adequate access to the tumor while minimizing potential damage to critical nerve structures.^5^,^12^,^13^ The majority of patients (94.3%) underwent gross-total, near-total and subtotal resection while only a few required biopsy due to lesion characteristics. This preference for excision reflects the surgical aim of achieving maximal tumor removal and decompression of the spinal cord, while balancing operative risks such as increased duration and blood loss.

Postoperative outcomes in terms of symptom relief demonstrated a mixed pattern. Immediate relief was reported in 57.1% of patients, with delayed improvement in a further subset. However, 25.7% of patients did not report symptomatic relief, primarily those undergoing subtotal resections or minimal interventions. Such outcomes highlight the challenge posed by residual tumors or tumor-related neurological deficits, necessitating closer surveillance and possible adjuvant therapies, as demonstrated with radiotherapy for specific lesions like those with subtotal resection or recurrent tumors.^12^,^13^

The types of complications encountered were diverse, with some patients suffering new neurological deficits and cerebrospinal fluid (CSF) leaks. Our study showed 5.7% (n=2) incidence of CSF leaks, primarily in cases of subtotal resection or biopsy, managed through re-exploration, dural defect repair with a fascial patch, and sealant, In contrast, Owen P. Leary’s study focused on wound complications rather than CSF leaks but he acknowledged that negative pressure wound therapy (NPWT) is traditionally avoided in such cases due to concerns about worsening the leak.^15^

The anatomical site of the tumors was predominantly located around the D12 to L1 levels, corroborating findings from existing studies that indicate a high incidence of tumors in this vulnerable spinal segment. This area is not only mechanically stressed due to its transitional nature between the rigid thoracic and more mobile lumbar sections but is also susceptible to both traumatic injuries and degenerative conditions that can predispose the area to tumor formation.^5^,^6^,^12^

This discussion emphasizes the multifaceted nature of managing spinal tumors at the dorsolumbar junction. The demographic data raise potential questions regarding preventive strategies; the confirmed predominance of intradural lesions reinforces the necessity of prompt and precise imaging protocols; surgical approaches should balance effective tumor resection with the minimization of complication risks; postoperative care requires tailored follow-up strategies to gauge therapeutic efficacy; and keen observation for potential tumor recurrence is essential for ongoing patient management. The cumulative knowledge emerging from this study serves not only as a foundation for local practices but also contributes to the broader discourse on spinal tumor management.

### Limitations:

This retrospective, single-center study from a Pakistani tertiary hospital included 35 patients and used non-probability sampling, which together with the narrow geographic catchment limits generalizability. Reliance on hospital records introduces potential selection and information bias, and the absence of long-term follow-up precludes robust assessment of durability, recurrence, and functional outcomes. All cases were managed via posterior approaches; alternative surgical strategies were not evaluated, introducing potential treatment-selection bias.

### Clinical Recommendations:

Surgical management should aim for gross total resection using posterior approaches, with careful neurological monitoring. Postoperative care must include regular follow-up, recurrence surveillance, and personalized rehabilitation strategies, ideally coordinated through a multidisciplinary tumor board to ensure optimal patient outcomes.

## CONCLUSION

This retrospective, descriptive series from a Pakistani tertiary center characterizes dorsolumbar junction (D11–L2) tumors, showing a predominance of intradural-extramedullary lesions—mainly neurofibromas and schwannomas—most often at D12–L1/L1–L2. A standardized posterior approach enabled gross/near-total resection in most cases with low complication rates and early symptomatic improvement. These results, achieved with MRI-guided planning and modern microsurgical tools, support that centers in Pakistan can deliver management comparable to international standards. As the study focuses on profile and early outcomes rather than head-to-head technique comparisons, larger multicenter Pakistani studies with longer follow-up are warranted to refine prognostic estimates and guide practice.

### Authors’ Contribution:

**TS:** Concept and design of the study, drafted the manuscript.

**AR, AK, NA:** Data acquisition and data analysis, drafted the manuscript.

**SM and SAD:** Supervision, critical revision of manuscript and data interpretation.

All authors agree to final approval of the version to be published be accountable for all aspects of the work.

## References

[1] Hafeez-ur-Rehman, Yousaf A, Usman M, Akram MN, Habib I, Ahmad S (2024). Positive predictive value of magnetic resonance imaging in intradural spinal tumors taking histopathology as gold standard. Pak J Neurol Surg.

[2] Dasarju VK, Sree S, Kikkeri MS, Shireesha B, Pallavi N, Kumar CS (2020). Magnetic resonance imaging in spinal tumors. Int J Contemp Med Surg Radiol.

[3] Li ST, Zhang T, Shi XW, Liu H, Yang CW, Zhen P (2022). Lumbar disc sequestration mimicking a tumor:report of four cases and a literature review. World J Clin Cases.

[4] Lee SH, Cha YJ, Cho YE, Park M, Joo B, Suh SH (2023). Clinicoradiologic characteristics of intradural extramedullary conventional spinal ependymoma. J Korean Soc Radiol.

[5] Sharma GR, Khadka N, Jha R, Adhikari DR, Bista P (2016). Extradural spinal tumors:report of 36 cases and review of literature. Nepal J Neurosci.

[6] Groenen KHJ, van der Linden YM, Brouwer T, Dijkstra SPD, de Graeff A, Algra PR (2018). The Dutch national guideline on metastases and hematological malignancies localized within the spine;a multidisciplinary collaboration towards timely and proactive management. Cancer Treat Rev.

[7] Kim SK, Bendardaf R, Ali M, Kim HA, Heo EJ, Lee SC (2022). Unilateral biportal endoscopic tumor removal and percutaneous stabilization for extradural tumors:technical case report and literature review. Front Surg.

[8] Rao S, Sugur H, Konar S, Arivazhagan A, Santosh V (2023). MYCN amplification in spinal ependymoma:a five-year retrospective study. Neuropathology.

[9] Kotecha R, Mehta MP, Chang EL, Brown PD, Suh JH, Lo SS, Das S (2019). Updates in the management of intradural spinal cord tumors:a radiation oncology focus. Neuro Oncol.

[10] Sarica FB, Morgan LR, Sarica FB (2019). Surgical principles for spinal and paraspinal neurofibromas. Brain and spinal tumors - primary and secondary.

[11] Patnaik S, Jyotsnarani Y, Uppin SG, Susarla R (2016). Imaging features of primary tumors of the spine:a pictorial essay. Indian J Radiol Imaging.

[12] Patel P, Mehendiratta D, Bhambhu V, Dalvie S (2021). Clinical outcome of intradural extramedullary spinal cord tumors:a single-center retrospective analytical study. Surg Neurol Int.

[13] Hachicha A, Belhaj A, Karmeni N, Slimane A, Bouali S, Kallel J (2021). Intramedullary spinal cord tumors:a retrospective multicentric study. J Craniovertebr Junction Spine.

[14] Shah LM, Salzman KL (2011). Imaging of spinal metastatic disease. Int J Surg Oncol.

[15] Leary OP, Setty A, Gong JH, Ali R, Fridley JS, Fisher CG (2025). Prevention and management of posterior wound complications following oncologic spine surgery:narrative review of available evidence and proposed clinical decision-making algorithm. Global Spine J.

